# Preimaginal Stages of the Emerald Ash Borer, *Agrilus planipennis* Fairmaire (Coleoptera: Buprestidae): An Invasive Pest on Ash Trees (*Fraxinus*)

**DOI:** 10.1371/journal.pone.0033185

**Published:** 2012-03-16

**Authors:** M. Lourdes Chamorro, Mark G. Volkovitsh, Therese M. Poland, Robert A. Haack, Steven W. Lingafelter

**Affiliations:** 1 United States Department of Agriculture, Agricultural Research Service, Systematic Entomology Laboratory, Washington, D.C., United States of America; 2 Laboratory of Insect Systematics, Zoological Institute, Russian Academy of Sciences, Saint Petersburg, Russia; 3 United States Department of Agriculture, Forest Service, Northern Research Station, East Lansing, Michigan, United States of America; University of Kentucky, United States of America

## Abstract

This study provides the most detailed description of the immature stages of *Agrilus planipennis* Fairmaire to date and illustrates suites of larval characters useful in distinguishing among *Agrilus* Curtis species and instars. Immature stages of eight species of *Agrilus* were examined and imaged using light and scanning electron microscopy. For *A. planipennis* all preimaginal stages (egg, instars I-IV, prepupa and pupa) were described. A combination of 14 character states were identified that serve to identify larvae of *A. planipennis*. Our results support the segregation of *Agrilus* larvae into two informal assemblages based on characters of the mouthparts, prothorax, and abdomen: the *A. viridis* and *A. ater* assemblages, with *A. planipennis* being more similar to the former. Additional evidence is provided in favor of excluding *A. planipennis* from the subgenus *Uragrilus*.

## Introduction

The emerald ash borer (EAB), *Agrilus planipennis* Fairmaire (Coleoptera: Buprestidae), is a metallic wood-boring beetle indigenous to eastern Asia, including China (Beijing, Hebei, Heilongjiang, Inner Mongolia/Nei Mongol, Jilin, Liaoning, Shandong, Sichuan, Tianjin, and Xinjiang); Taiwan; Japan; Korea; Mongolia; and Russian Far East [1], [2], [3]. In addition, Jendek and Grebennikov [4] state that *A. planipennis* occurs in Laos. In China, *A. planipennis* typically causes only minor damage to native tree species, generally attacking weakened or dying Asian ash (*Fraxinus* spp., Lamiales: Oleaceae) such as *Fraxinus chinensis* Roxb., *F. mandshurica* Rupr., and *F. rhychophylla* Hance [2], [3]. However, *A. planipennis* readily infests and kills both stressed and healthy North American ash species including *F. americana* L., *F. pennsylvanica* Marshall, and *F. velutina* Torr. when planted in China [5], [6] and has become one of the most serious invasive insect pests killing tens of millions of healthy ash trees in Eastern North America since its discovery in 2002 [1], [7], [8], [9] and in Moscow, Russia [10]. It has been estimated that between the years 2009–2019, 17 million landscape ash trees in urban areas across 25 states will require treatment, removal and replacement at a cost of approximately $10.7 billion [11]. The large-scale mortality now occurring to native ash in forested and urban settings in North America will undoubtedly change urban landscapes and impact forest system processes, including threatening many other insect taxa with close evolutionary and ecological ties to ash [12]. Besides ash trees, *A. planipennis* was reported to feed more rarely on *Juglans mandshurica* Maximowicz, *Pterocarya rhoifolia* Siebold & Zuccarini (Fagales: Juglandaceae) and *Ulmus davidiana* Planchon (Rosales: Ulmaceae) in Asia [1], [13]. In Europe, there is great concern that *A. planipennis* will spread westward from Moscow and threaten European ash species such as *F. angustifolia* Vahl, *F. excelsior* L., and *F. ornus* L. [10], [13], [14], [15].

The higher levels of resistance demonstrated by Asian ash species to *A. planipennis* as compared with European and North American ash species is likely related to the fact that Asian ash species co-evolved with *A. planipennis*, while those in Europe and North America did not [16]. The evolutionary arms-race [17] between the wood-boring *A. planipennis* and its native Asian ash hosts has allowed Asian ashes to develop a suite of physical and phytochemical defenses that protect the trees against *A. planipennis* infestation except during times of environmental stress such as drought [18]. However, the non-Asian ash species lack these resistance mechanisms and thus are easily infested by *A. planipennis* even when healthy. A similar situation occurs in the case of *Agrilus anxius* Gory (bronze birch borer), a North American birch (*Betula* spp., Fagales: Betulaceae)–infesting species, that is usually only capable of infesting stressed North American birch, but can easily infest and kill European and Asian birch when planted in North America [19].

An effort is currently underway to identify relatives of *A. planipennis* that may pose a risk to North American woody plants if accidentally introduced [20]. Increased knowledge of larval morphology, along with a sound understanding of basic biology and ecology should help elucidate key evolutionary adaptations that allow some *Agrilus* Curtis species to become highly invasive when introduced to new environments. Of particular interest are adaptations that contribute to the ability of *A. planipennis* to effectively attack and kill healthy ash trees and undermine their defenses.

Currently, 34 informal species-groups [4], [21] and 36 subgenera, based mainly on Palearctic species and the adult stage, are recognized in *Agrilus* [4]. Alexeev [22] placed *A. planipennis* in the subgenus *Uragrilus* Semenov together with *A. ater* (Linnaeus), *A. guerini* Lacordaire, *A. sachalinicola* Obenberger, *A. tscherepanovi* Stepanov ( =  *A. fleischeri fleischeri* Obenberger) (Palearctic), *A. acutipennis* Mannerheim, *A. anxius*, *A. bilineatus* (Weber), *A. quadriguttatus* Gory, *A. quadriimpressus* Ziegler, *A. ruficollis* (Fabricius), *A. vittaticollis* (Randall) (Nearctic), and *A. rokuyai* Kurosawa (Oriental). Use of this classification has been correctly criticized because it is based on a limited sample of known *Agrilus* diversity [4], [23], however it remains largely unchanged pending comprehensive taxonomic and phylogenetic studies.

**Figure 1:**
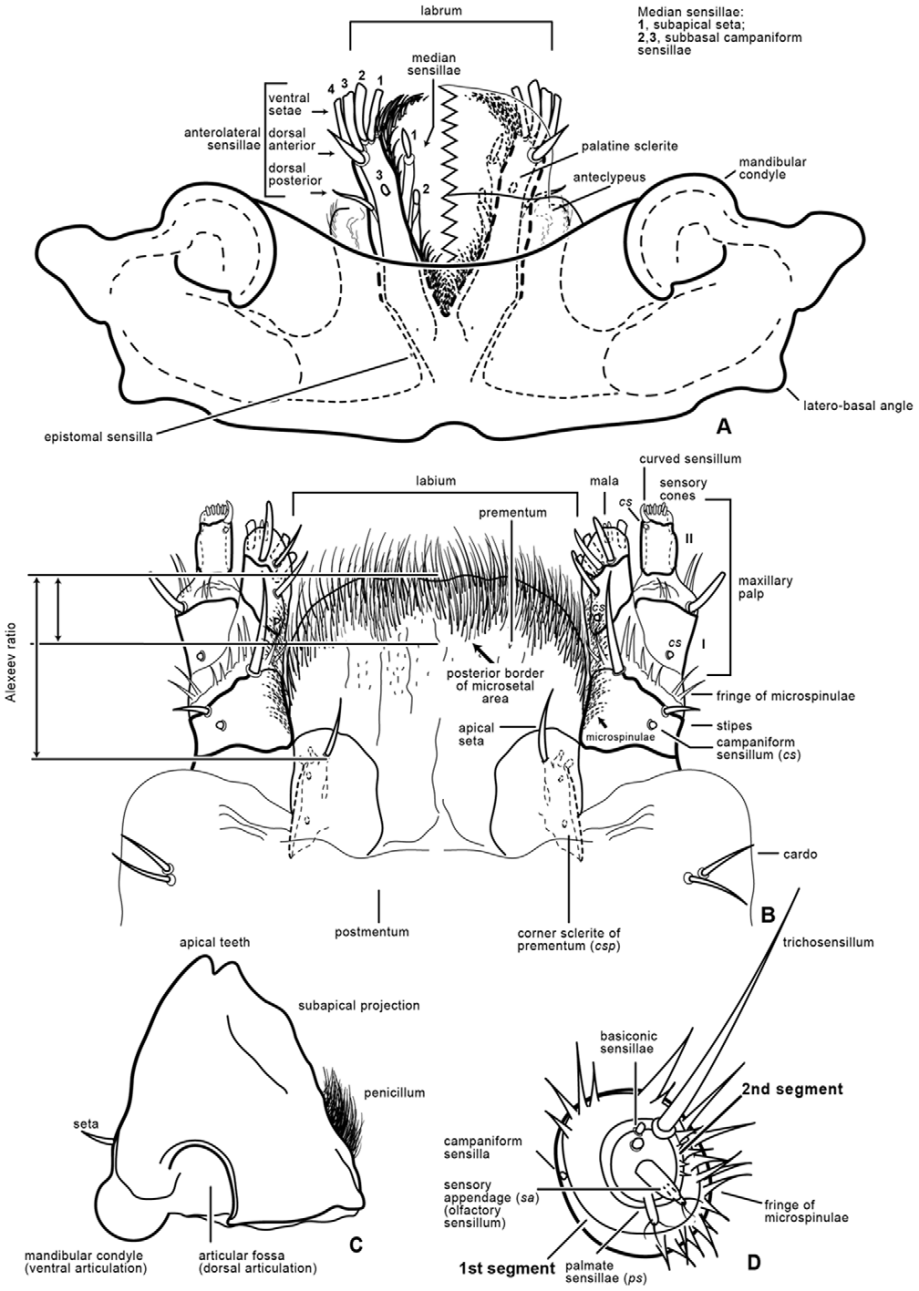
Terminology used for mouthparts and antennae, *Agrilus planipennis*. A, epistome; B, labio-maxillary complex; C, mandible (left); D, antenna, anterior view.

**Figure 2:**
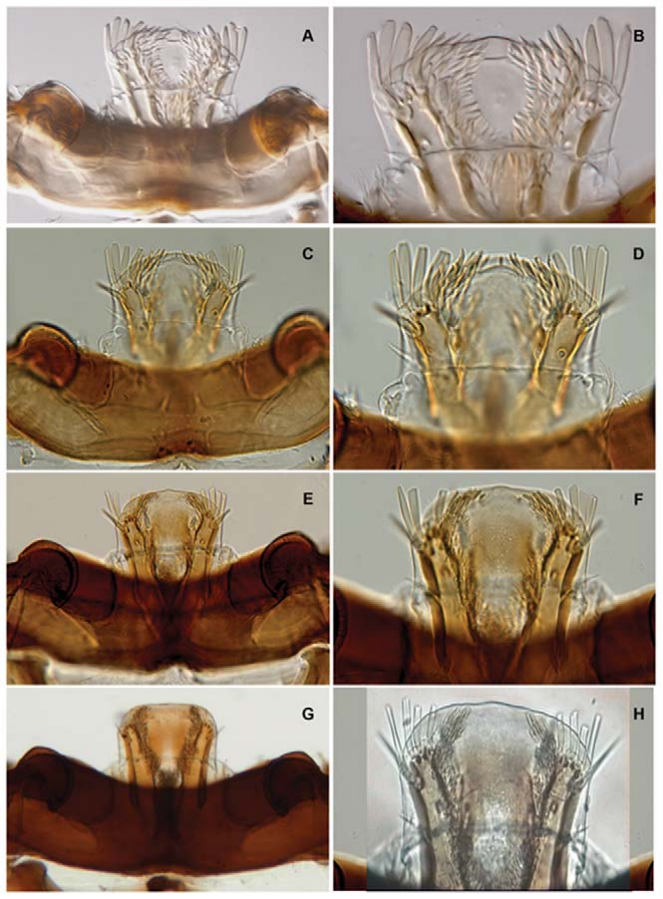
Epistome, labrum and palatine sclerites of *Agrilus planipennis* instars I, II, III, IV. A, instar I, epistome and labrum; B, instar I, labrum and palatine sclerites; C, instar II, epistome and labrum; D, instar II, labrum and palatine sclerites; E, instar III, epistome and labrum; F, instar III, labrum and palatine sclerites; G, prepupa, epistome and labrum; H, instar IV, labrum.

**Figure 3:**
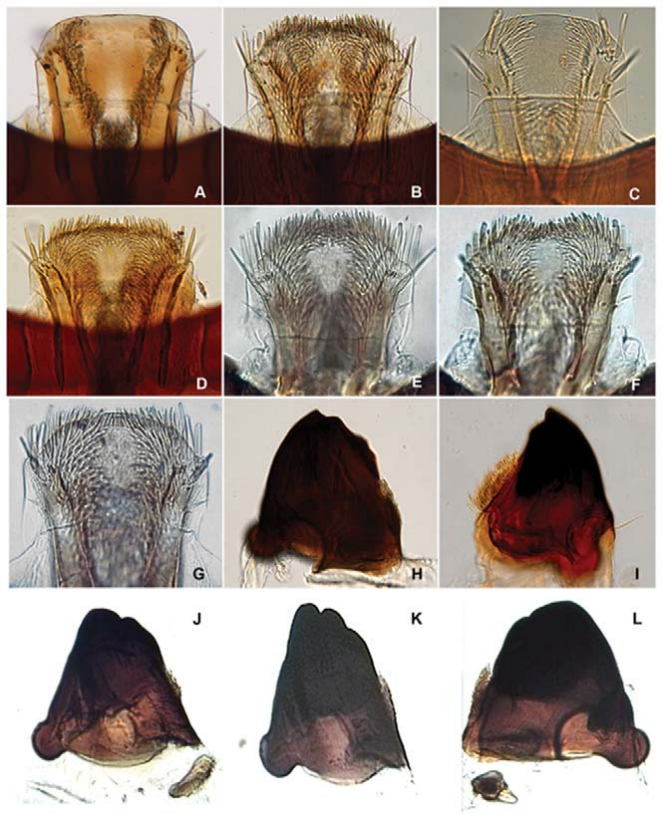
*Agrilus* larvae labrum and mandibles. A, *Agrilus planipennis*, labrum; B, *Agrilus anxius*, labrum; C, *Agrilus politus*, labrum; D, *Agrilus guerini*, labrum; E, *Agrilus ater*, labrum; F, *Agrilus biguttatus*, labrum; G, *Agrilus australasiae*, labrum; I, *Agrilus anxius*, mandible; I, *Agrilus guerini*, mandible; J, *Agrilus biguttatus*, mandible; K, *Agrilus ater*, mandible; L, *Agrilus australasiae*, mandible.

**Figure 4:**
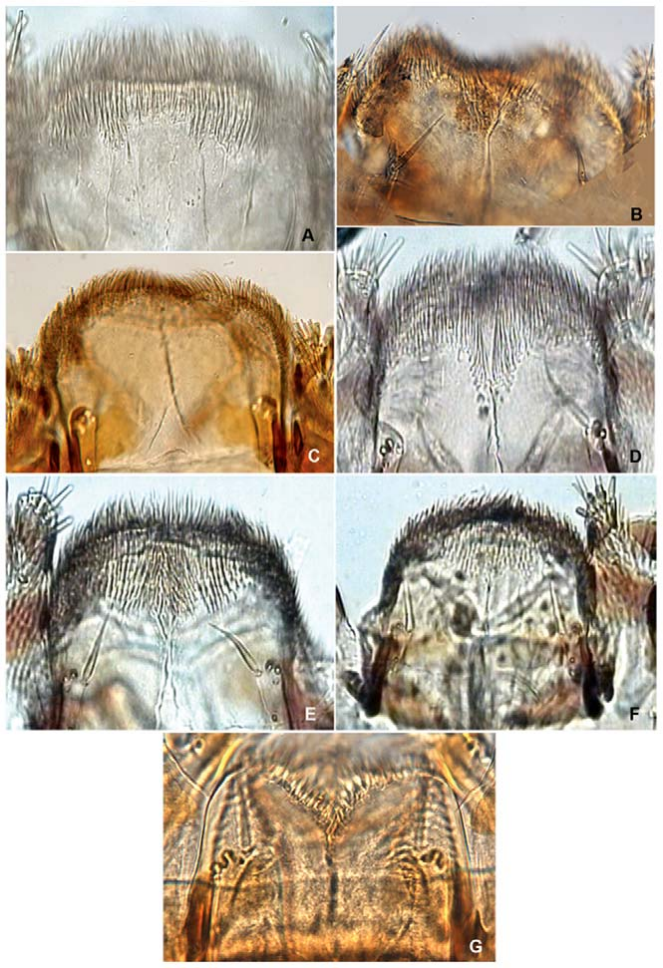
Agrilus larvae prementum. A, Agrilus planipennis; B, Agrilus anxius; C, Agrilus guerini; D, Agrilus ater; E, Agrilus australasiae; F, Agrilus biguttatus; G, Agrilus politus.

**Figure 5:**
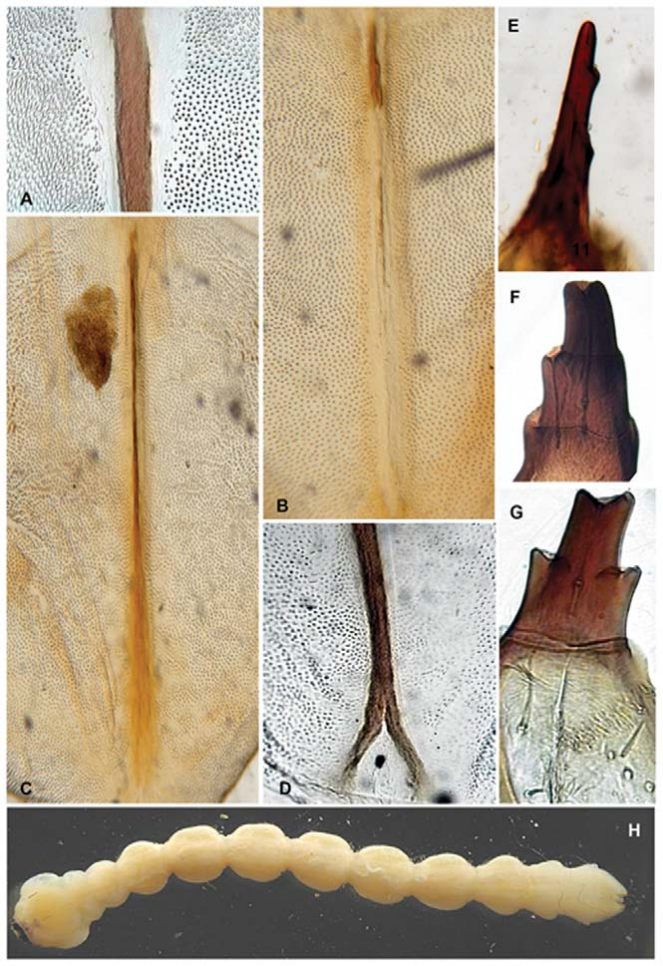
*Agrilus* larvae thoracic grooves, terminal processes, and *Agrilus nubeculosus* habitus. A, *Agrilus australasiae*, detail of pronotal groove; B, *Agrilus guerini*, prosternal groove; C, *Agrilus guerini*, pronotal groove; D *Agrilus biguttatus*, detail of pronotal groove; E, *Agrilus guerini*, terminal processes; F, *Agrilus australasiae*, terminal processes; G, *Agrilus biguttatus*, terminal processes; H, *Agrilus nubeculosus*, dorsal habitus, instar IV.

Volkovitsh & Hawkeswood [24] segregated *Agrilus* larvae into two informal groups or assemblages based on 1) presence or absence of microsetal areas along the anterior margin of the labrum and 2) of distinct zones of microspinulae concentrated on the internal surface of the maxillae (Figure 1): the *A. viridis* species-assemblage and *A. ater* species-assemblage. Based on their study, Volkovitsh & Hawkeswood [24] included in the *A. viridis* assemblage the following species: *A*. (*Agrilus*) *viridis* (Linnaeus) (Palearctic) (the type species of the genus); *A.* (*Agrilus*) *ribesi* Schaefer (Palearctic); *A*. (*Agrilus*) *cuprescens* (Ménétries) (Palearctic/Nearctic); *A*. (*Quercuagrilus*) *sulcicollis* Lacordaire (Palearctic; recently introduced to Canada and USA [25], [26]); A. (*Quercuagrilus*) *hastulifer* Ratzeburg (Palearctic); and *A*. (*Quercuagrilus*) *angustulus* (Illiger) (Palearctic). Species in the *A*. *viridis* assemblage have a glabrous anterior margin of the labrum (Figures 1, 2, 3), fringe of microspinulae between maxillary stipes and base of maxillary palpus, and microspinulae concentrated subapically on the mala and internal surface of the stipes and cardo (Figure 1b) [24]. Species included by Volkovitsh & Hawkeswood [24] in the *A*. *ater* assemblage were: *A.* (*Uragrilus*) *ater* (Linnaeus, 1767) (Palearctic) (Figures 3e, 3k, 4); *A.* (*Agrilus*) *australasiae* Laporte & Gory (Australasian) (Figures 3g, 3l, 4e, 5); and *A.* (*Anambus*) *biguttatus* (Fabricius) (Palearctic) (Figures 3f, 3j, 4f, 5d, 5g). These species have a dense microsetal/microspinulated area on the anterior margin of the labrum, the epipharynx (Figures 3b, 3d–3g), and the internal surface of the maxillae, more than species in the *A. viridis* assemblage.

Despite its economic importance as an invasive species, all preimaginal stages, which include egg, instars I-IV, prepupa (non-feeding terminal phase of instar IV), and pupa, of *A. planipennis* remain superficially described. Generalized descriptions of the larvae have been included in various biological or ecological studies [2], [3], [27], [28]. Moreover, detailed descriptions of *Agrilus* larvae have been reported for only a small number of species in the genus [24], [29], [30], [31], [32], [33], [34], [35]. Generalized information on biology and morphology of mostly Palearctic [34], [36], [37], [38] and North America *Agrilus* species [39], [40] is more common. With more than 2,750 species [21] recognized in the genus *Agrilus*, it remains a monumental task to amass descriptions and life history data for all *Agrilus* species worldwide. This study presents the first detailed description of the egg, larval instars I–IV, prepupa, and pupa of *A. planipennis* and compares the larvae to 7 *Agrilus* species to determine its affinity.

## Results

### Description

We present a detailed morphological description for instar IV of *Agrilus planipennis*, along with egg, instars I-III, prepupa, and pupa. Since overall morphology is very similar between all instars, we describe only important distinguishing characteristics for stages I-III and prepupa for brevity. See ‘Discussion’ for more elaboration.

### Agrilus planipennis Fairmaire

#### Instar IV

**Figure 6:**
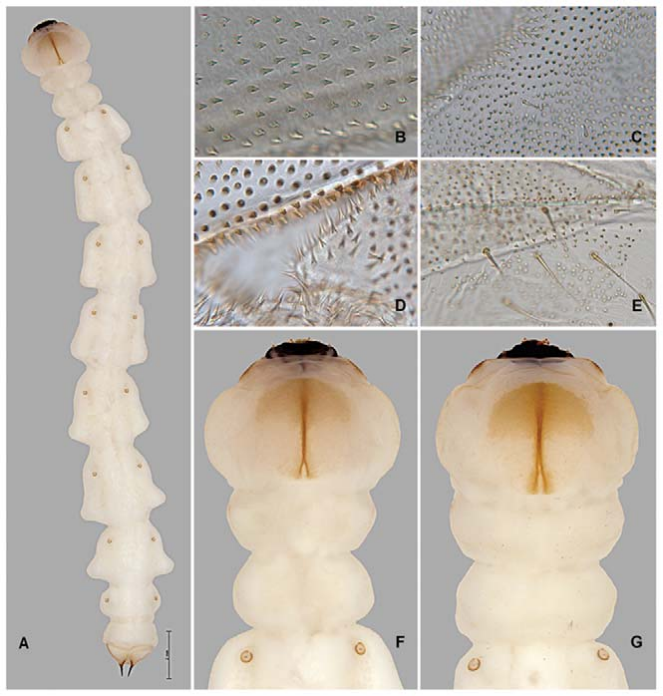
*Agrilus planipennis* instar IV including prepupa. A, instar IV, dorsal view; B, prepupa, thoracic microspinulae; C, prepupa, thoracic microspinulae and setae; D, prepupa, microdenticles (top), microsetae (bottom); E, prepupa, pleural region of abdomen, setae and microspinulae; F, instar IV, dorsal view, detail of peristome, pro-, meso-, metathorax, and 1st abdominal segment with spiracles; G, prepupa, same as F.

**Figure 7:**
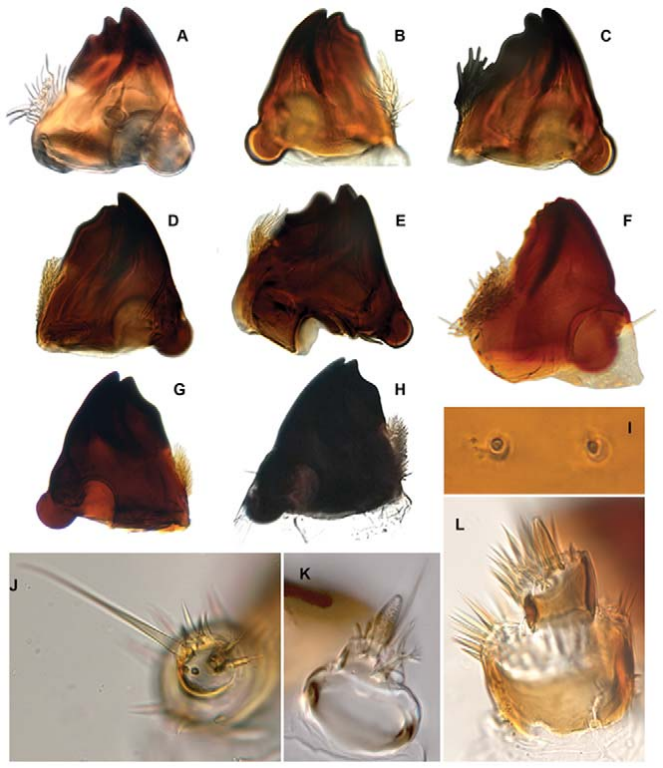
*Agrilus planipennis* larval mandibles, antennae, epistomal sensory pits, and *Agrilus politus* larval mandible. A, instar I, left mandible; B, instar II, right mandible; C, instar II, left mandible; D, instar III, left mandible, E, instar III, right mandible, oblique–lateral view; F, prepupa, right mandible; G, instar IV, right mandible; H, *Agrilus politus*, left mandible; I, instar I, epistomal sensory pits; J, prepupa antenna, anterior view; K, instar I, antenna, lateral view; L, prepupa antenna, lateral view.

**Figure 8:**
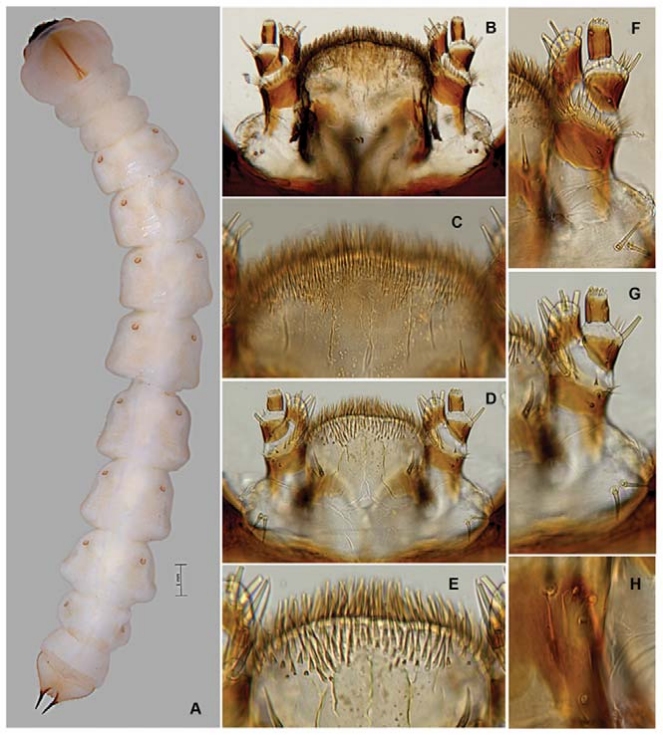
*Agrilus planipennis* habitus and labio-maxillary complex. A, prepupa, habitus, dorsal view; B, prepupa, labio-maxillary complex; C, prepupa, detail of labium and setal area; D, instar III, labio-maxillary complex; E, instar III, detail of labium and setal area; F, prepupa, maxillae; G, instar III, maxillae; H, prepupa, corner sclerite of prementum (top), campaniform sensilla.

**Figure 9:**
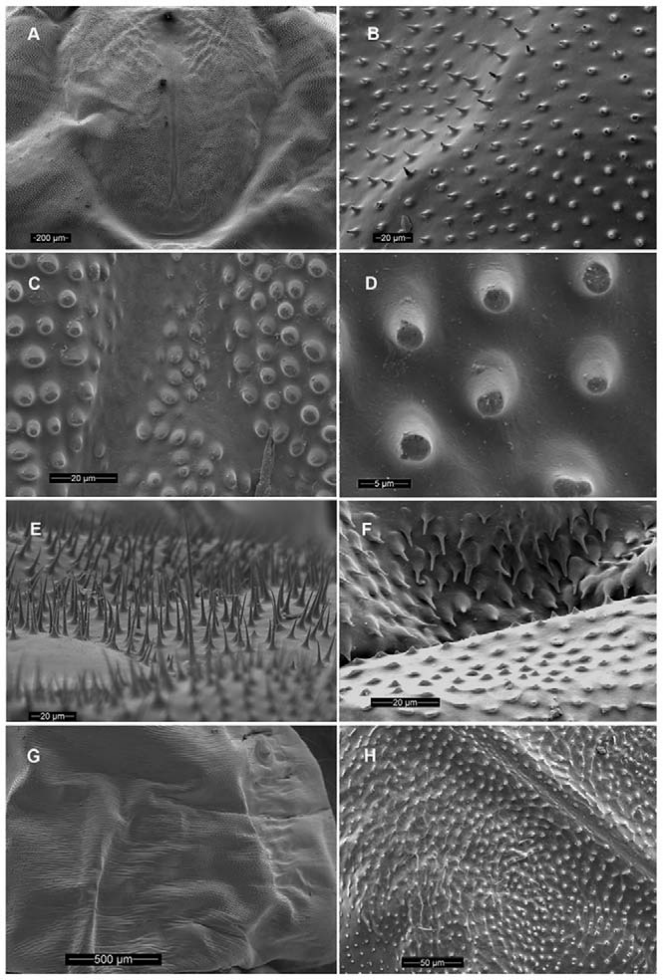
Scanning Electron Micrographs of *Agrilus planipennis* larvae. A, prepupa, pronotum; B, prepupa, detail of pronotum, side; C, prepupa, detail of divergence of pronotal groove; D, prepupa, detail of microdenticles near pronotal groove; E, prepupa, microsetae posterad of mouthparts; F, instar II, anterior edge of pronotum; G, prepupa, abdominal segment I, dorsal view; H, instar II, prosternum.

**Figure 10:**
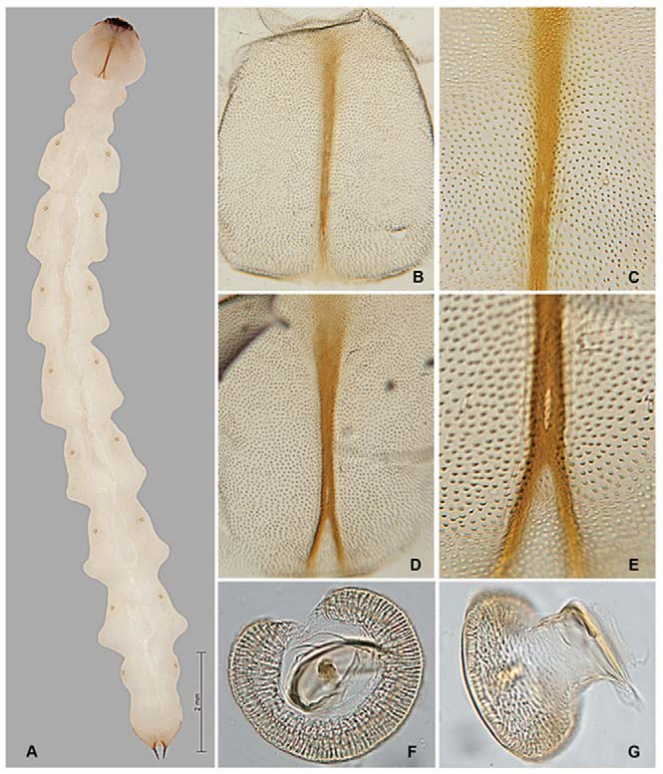
*Agrilus planipennis* instar III, habitus, thoracic grooves, and spiracles. A, dorsal view; B, prosternal plate and groove; C, detail of prosternal groove, note microsetae; D, pronotal plate and groove; E, detail of prosternal groove; F, spiracle, anterior view; G, spiracle, lateral view.

**Figure 11:**
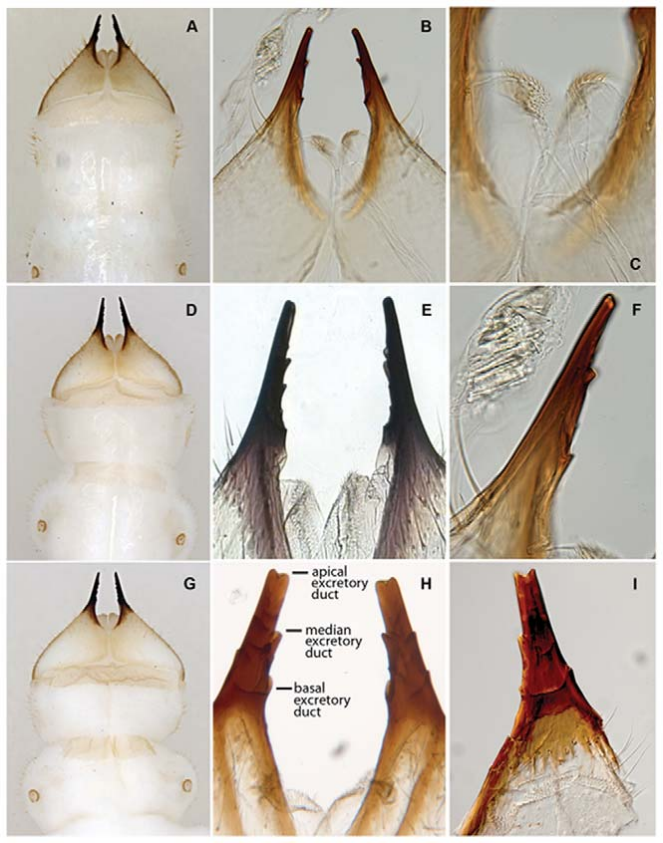
*Agrilus planipennis* larval terminal processes. A, instar III, dorsal view; B, instar III, detail of terminal processes; C, instar III, detail of anal opening; D, instar IV, dorsal view; E, instar IV, detail of terminal processes; F, instar IIII, detail of single terminal process; G, prepupa, dorsal view; H, prepupa, detail of terminal processes; I, instar IV, detail of single terminal process.

**Figure 12:**
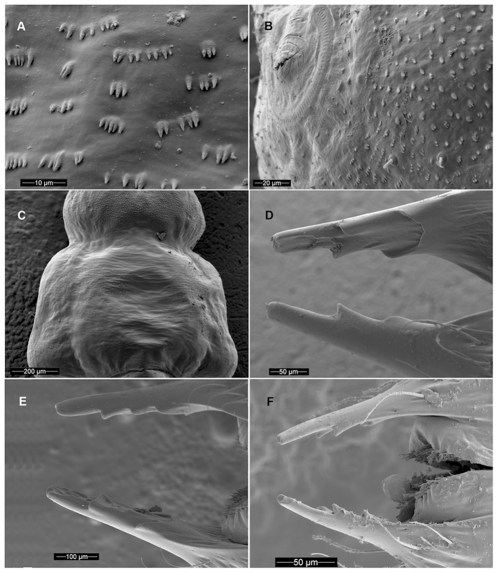
Scanning Electron Micrographs of *Agrilus planipennis* larvae. A, instar II, detail of microspinulae on abdominal segment 2, ventro-lateral; B, instar II, detail of mesothoraxic left spiracle and microspinulae, ventral; C, instar II, abdominal segments 1 and 2, ventral showing patches of lateral microspinulae, ventral; D, instar III, terminal processes, oblique lateral view; E, prepupa, terminal processes, dorsal view; F, instar II terminal processes, dorsal view.

Length 30–36 mm. Larva of typical agrilinoid type with enlarged prothoracic and abdominal segments 1–7 and heavily sclerotized paired terminal processes (Figure 6). Body whitish with yellowish prothoracic plate, highly pigmented (brown) peristome, prothoracic (notal and sternal) grooves, spiracles, and terminal processes. Head and mouthparts. Epistome (Figure 1a) strongly transverse, 3.5–5.5 times wider than long; bearing 2 pairs of epistomal sensillae medially, arranged one directly ventral to the other, each pair consists of single anterior sensillum and 2 sunken posterior basiconic sensillae in the common pit (Figures 1a, 7); anterior margin concave between paired mandibular condyles; posterior margin bisinuate; latero-basal angles prominently rounded, blunt. Anteclypeus (Figures 1a, 2h, 3a) narrow, membranous, glabrous, anterior margin almost straight. Labrum (Figures 1a, 2h, 3a) elongate, 1.5 times longer than wide, anterior margin widely arcuate and glabrous, lateral margins subparallel, mostly membranous bearing distinct paired palatine sclerites, each divided into medial and lateral subparallel branches and slightly divergent from longitudinal midline of labrum; median sensillae of labrum along medial branches consist of a long subapical seta and 2 subbasal campaniform sensillae situated subequally (Figure 1a); anterolateral sensillae (Figure 1a) of each palatine sclerite with long dorsal anterior and short dorsal posterior seta, 4 flattened blunt anterior ventral setae arranged linearly. Epipharynx with microsetae situated only along median branches with central and lateral parts glabrous (Figure 1a). Paired antennae (Figures 1d, 7j, 7l) each 2-segmented, 1^st^ segment subcylindrical, sclerotized, about 1.5 times wider than long, with campaniform sensillum on lower half of internal lateral margin and fringe of microspinulae around apex (anterior margin); 2^nd^ segment as long as wide, subcylindrical with very long, sharp trichosensillum, prominent sensory appendage (*sa*), 2 palmate sensillae (*ps*), 2 basiconic sensillae at base of sensory appendage and tuft of long microspinulae apically. Paired mandibles (Figures 1c, 7g) each triangular, heavily sclerotized, bearing 2 apical teeth and subapical projection, internal margin with large penicillum consisting of elongate microtrichia, short external seta adjacent to condyle (sometimes broken off). Labio-maxillary complex (Figures 1b, 8): Paired maxillae (Figures 1b, 8f, 8g) each with cardo completely membranous with laterobasal sclerite absent, only 2 setae on membrane; stipes (Figure 1b) moderately sclerotized, long seta at base of mala, campaniform sensillum (*cs*) and seta laterally, fringe of microspinulae along anterior margin (Figure 8f). Paired maxillary palpi (Figure 1b) each 2-segmented, 1st segment about as long as wide, with long, sharp seta arising near anterolateral margin and campaniform sensillum almost below long seta closer to base, anterior margin glabrous with group of microspinulae at anterolateral corner; 2^nd^ segment about 2 times longer than wide, heavily sclerotized, with campaniform sensillum medially along external (lateral) margin, curved (digitiform) sensillum along internal margin, apically 7–8 small, sensory cones. Paired mala (Figures 1b, 8f, 8g) strongly sclerotized, about 1.5 longer than wide, basiconic sensillum medially, 2–3 thick setae externally and 5 large, thick, mostly blunt setae internally with numerous microspinulae present. Labium (Figures 1b, 4a, 8c, 8e): prementum transverse about as long as wide with widely arcuate anterior margin; externally (ventrally) with dense microsetae forming microsetal area along anterior margin, posterior border of this area zigzag-shaped, extending about 1/3 distance from anterior margin to base of apical seta of paired corner sclerites of prementum (Figure 1b, *csp*); each corner sclerite bearing basal and 4–5 small apical campaniform sensilla (Figure 8h), short apical seta not extending to posterior border of microsetal area (Figure 4a). Hypopharynx with microsetae along anterolateral corners. Postmentum (Figure 1b) glabrous. Thorax (Figures 6f, 9). Prothorax approximately as wide as abdominal segments 1–7, meso- and metathorax each slightly narrower than prothorax (Figures 6f, 6g). Prothoracic plate pigmented, anterior half darker, round, completely covered with heavily sclerotized microdenticles situated on small rounded tubercles changing to small microspinulae toward periphery of plate (Figures 6b, 6d top, 10), with sparse short setae (Figures 10d, 10e). Pronotal groove (Figures 6f, 6g, 10d, 10e) very distinct, dark brown, bifurcating from almost posterior 1/5. Prosternal groove (Figures 10b, 10c) very distinct, dark brown, fragmenting anteriorly, with surrounding microdenticles more heavily sclerotized, asperate, remaining dark (Figure 10c). Pro-, meso-, and metathorax with microspinulae, laterally with microspinulae and setae (Figure 6e). Pair of thoracic spiracles anterolaterally on mesothorax (not visible from above), of the agriloid circular type (Figures 10f, 10g), heavily sclerotized, cribriform with relatively short, spiracular trabeculae. Thoracic and abdominal spiracles similar, differing in size and trabeculae number. Abdomen (Figure 6a). Abdominal segments pale cream colored, segments 2–7 becoming increasingly trapezoidal, almost bell-shaped (predominantly 5–7), of approximately equal width and length, segment 1 almost 1/3 shorter than subsequent segments, segments 8–9 about half as tall as preceding, each narrower than preceding segment; segments 1–8 dorsally and ventrally bearing shallow longitudinal lateral depressions, dorsally situated almost posterad to spiracles; bottom of depressions covered with darker internal reticulation. Segment 10 (anal segment, Figures 11, 12) deltoid, bearing setae laterally, zones of microspinulae around anal opening; apically with heavily sclerotized paired terminal processes with apical, median, and basal excretory ducts present (Figures 11e, 11h, 11i, 12e) and numerous secondary subdivisions (ledges) (Figures 11e, 12e) [27]. Dorsal surface of abdomen almost glabrous, segments 1–9 with paired oval, subparallel bands of very light microspinulae, laterally with setae, microspinulae posterolaterad; segments 8–9 with transverse posterior zones of microspinulae. Paired spiracles anterodorsally on abdominal segments 1–8.

#### Pupa

**Figure 13:**
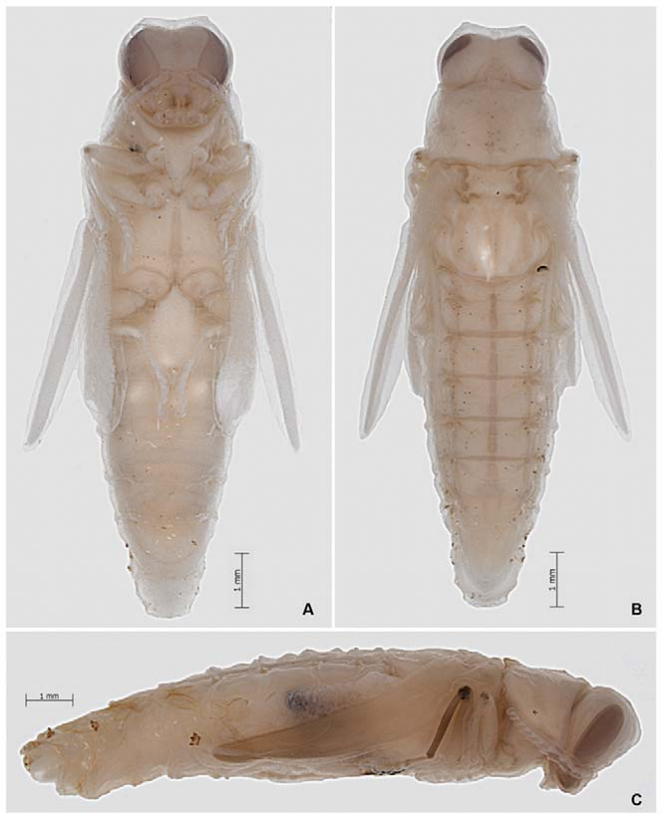
*Agrilus planipennis* pupa. A, ventral view; B, dorsal view; C, lateral view.

**Figure 14:**
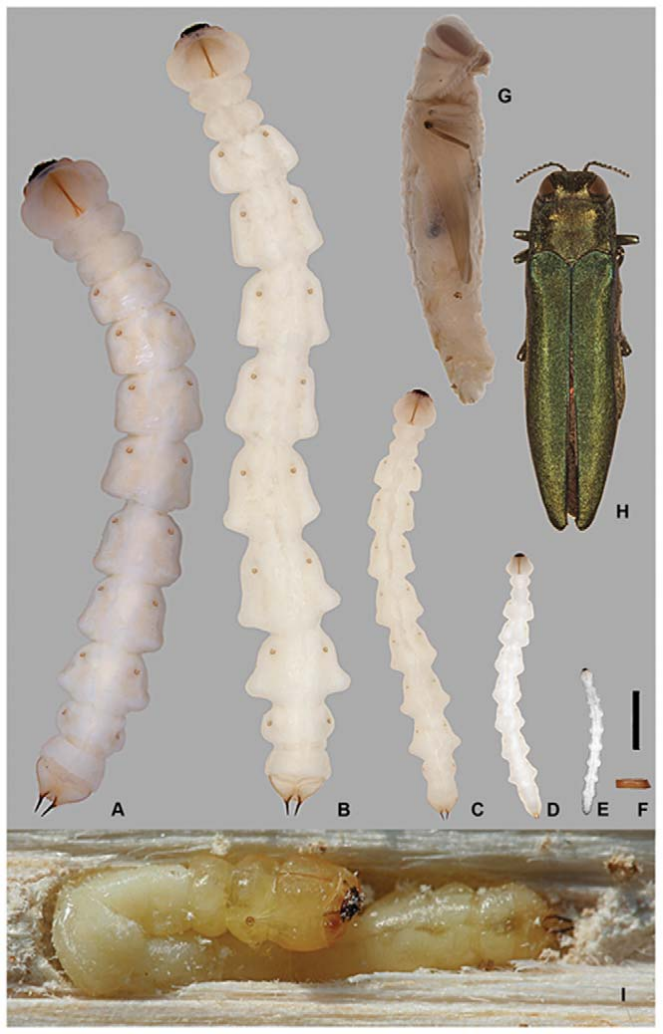
All stages of *Agrilus planipennis*. A, prepupa, B, instar IV; C, instar III; D, instar II; E, instar I; F, egg; G, pupa; H, adult; I, prepupa curled in chamber (photo UGA510033). Scale bar 2 mm.

Length 13.0–17.5 mm; width 4.0–5.5 mm. Adecticous, exarate; color whitish (Figures 13, 14). Head. Tilted posteroventrad with occiput exposed, in dorsal view; eyes large, dorsally separated by length larger than visible dorsum of eye; median eye margin sinuate, in anterior view; frons concave; antenna reaching base of prothorax. Thorax. Quadrate, parallel-sided. Scutellum rectangular, disc not differentiated; elytron reduced, elongate; metanotum quadrate, wings reduced, elongate and narrow. Abdomen. Eight segmented, spiracles dorsolaterally on segments 1–5.

#### Prepupa

Length 27–35 mm (Figures 2g, 6b–6e, 6g, 7f, 7j, 7l, 8a–8c, 8f, 8h, 11g, 11i, 12e, 14a, 14i). Abdominal and thoracic segments and intersegment space compacted (Figure 8a). Thorax. Meso- and metathorax subequal to prothorax; body recurved between abdominal segments 2 and 4 (Figure 14i); posterior third of pronotal groove bifurcated (Figure 6g). Abdomen. Sternites 1–9 with paired oval, subparallel bands of microspinulae more prominent and microspinulae darker posterolaterad than instar IV; lateral depressions indistinct. Terminal processes as in instar IV, with 3 excretory ducts and numerous ledges (Figure 12e).

#### Instar III

Length 16–26 mm (Figures 2e, 2f, 7d, 7e, 8d, 8e, 8g, 10a–11c, 11f, 12d, 14c). Head and Mouthparts. Mandibles strongly, nearly uniformly sclerotized (Figures 7d, 7e). Thorax and Abdomen. Microspinulae weaker than in instar IV; terminal processes longer than in instars I and II; apical, median, and basal excretory ducts present and minor ledges visible, mostly between median and apical ducts (**Figures 11a**–11b, 11f, 12d) [27].

#### Instar II

Length 10–12 mm (Figures 2c, 2d, 7b, 7c, 9h, 12a–12c, 12f, 14d). Body shape similar to instar I (Figures 14d, 14e). Head and Mouthparts. Mandibles more strongly sclerotized than instar I, light brown with darker apex (Figures 7b, 7c); fringe of microspinulae lacking around apex of antennal segment 1 (Figure 7k). Thorax and Abdomen. Microspinulae on thorax and abdomen serial, comb-like (Figures 12a–12c). Terminal processes shorter than in instar III apical, median, and basal excretory ducts present (Figure 12f) [27], [41]; subdivisions (ledges) absent.

#### Instar I

**Figure 15:**
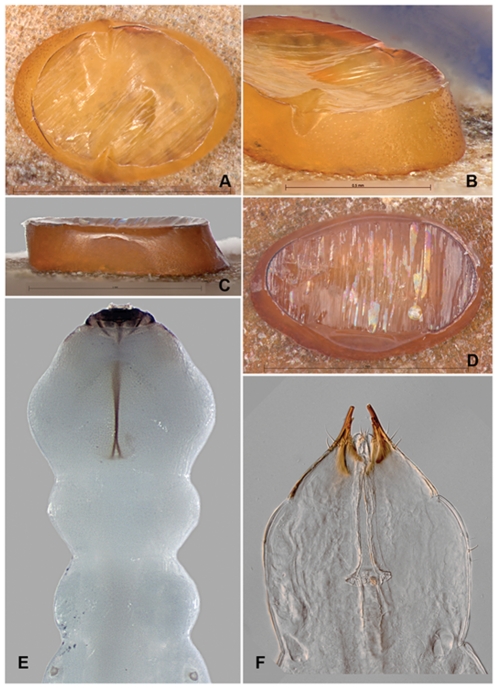
*Agrilus planipennis* egg and instar I. A, egg, dorsal view; B, egg, oblique lateral view; C, egg, lateral view; D, egg, dorsal view; E, instar I, mouthparts, pro-, meso-, metathorax, and anterior part of 1^st^ abdominal segment bearing spiracles, dorsal view; F, instar I, terminal processes, segments 8–10, dorsal view.

Length 6.6 mm (Figures 2a, 2b, 7a, 7i, 7k, 14e, 15). Head and Mouthparts. Antenna with nearly glabrous upper margins, microspinulae indistinct (Figure 7k). Labrum as wide as tall; ventral antero-lateral setae relatively large compared to labrum (Figures 2a, 2b); mandibles poorly sclerotized, yellowish with only apex dark (Figure 7a); fringe of microspinulae lacking around apex of antennal segment 1 (Figure 7k). Thorax and Abdomen. Microspinulae less pigmented and less prominent than in later instars (Figure 15e). Terminal processes shorter than instar II, apical and median excretory ducts present, subdivisions (ledges) absent (Figure 14f) [27].

#### Egg

Length 1.0–1.2 mm; height 0.3 mm; width 0.6 mm. Color yellowish-orange to orange-brown (Figures 14f, 15a–15d). External lateral surface porous–like; dorsally smooth and shiny with streaked gelatinous appearance, resembling plastic film from above; symmetric depressions medially on lateral margins of dorsum. Glue-like substance on venter of egg that helps it adhere to bark surface.

## Discussion

Our results provide additional evidence [1], [4], [42] in favor of excluding *A. planipennis* from the subgenus *Uragrilus*. The larvae of 3 additional species currently classified in *Uragrilus* were examined for this study: *A. anxius*, *A. ater*, and the type species of the subgenus, *A. guerini*. These 3 species fall within the *A. ater* assemblage *sensu* Volkovitsh & Hawkeswood [24] because they have a pubescent, laterally expanded labrum and maxillae with a dense covering of microspinulae on the internal surface. In fact, larvae of *A. anxius* resemble Palaearctic species of *Uragrilus*, but additional phylogenetic and comparative studies are required to confirm relatedness. Larvae of *A. planipennis* instead share more features with *A. politus* and other species in the *A. viridis* assemblage *sensu* Volkovitsh & Hawkeswood [24]. These species have a glabrous labrum and microspinulae concentrated subapically on the mala and internal surface of the stipes and cardo. This suggests that based on larval characters, *A. planipennis* does not belong in the subgenus *Uragrilus* as proposed by Alexeev [22] based on adult characters and its subgeneric position is unclear. Based on adult features, *A. planipennis* is considered to be more closely related to species in the *A. cyaneoniger* group [1], [4], but the immature stages of species in this group remain unknown.

Larval characters useful in species discrimination include: 1) overall shape of abdominal segments; 2) pigmentation of pronotal and prosternal grooves [30]; 3) shape of either groove (entire or bifurcated); 4) presence or absence of glabrous space surrounding either groove; 5) structure of terminal processes [30], including the number, shape, and size of the excretory ducts (invagination of the inner surface of the urogomphi *sensu* Petrice *et al.* [41]), and presence/absence of ledges, particularly in latter instars; 6) extent of pilosity and shape of anterior margin of labrum (glabrous or pubescent and margin shape); 7) setation of labial prementum, which includes the relative length of apical setae on the corner sclerites; distance between bases of apical setae to posterior border of microsetal area (Alexeev ratio, Figure 1b); shape of posterior border of microsetal area (i.e., arcuate, zigzag, truncate, etc.) and of entire setal labial area; 8) sclerotization, shape of apical teeth of the mandible, and size of penicillum (Figures 1c, 3i, 7h); 9) extent of pilosity, proportions, and shape of apical antennal segment; and 10) size, shape, and number of spiracular trabeculae. The characters and their states are described below:

1. The overall shape of each abdominal segment, more pronounced in posterior segments, of *A. planipennis* is trapezoidal or bell-shaped, having the posterolateral angles produced laterad (Figures 6a, 10a, 14) (less in the prepupa, Figure 8a), differing from other known *Agrilus* larvae which have individual subquadrate abdominal segments (Figure 5h). The function of trapezoidal abdominal segments remains unknown.
2–4. The pronotal groove of *A. planipennis* is posteriorly bifurcate (Figures 6f, 6g, 10d–10e) and lacks a smooth space or border surrounding the groove. A similar pronotal groove is found in other species such as *A. biguttatus* (*A. ater* assemblage) (Figure 5d), therefore a posteriorly bifurcated pronotal groove is not unique to *A. planipennis*. Alternatively, the pronotal groove in other species may be entire as in *A. politus* and *A. anxius*, or also bordered by a glabrous area as in *A. australasiae* (Figure 5a), and to a lesser extent in *A. guerini* (Figures 5b–5c). The prosternal groove is entire in the species examined, including *A. planipennis* (Figure 10b). Some species may have a short posterior bifurcation (e.g., *A. anxius*). A smooth area may border the prosternal groove as in *A. guerini* (Figure 5b), *A. anxius*, and *A. australasiae* (Figure 5a), but absent in *A. planipennis* (Figures 10b–10e) and *A. biguttatus* (Figure 5d). The extent of the smooth area and posterior bifurcation may differ among species.
5. The terminal processes of *A. planipennis* are long, cylindrical and narrow and surrounded by few setae. With each subsequent instar the terminal processes become longer and the number of subdivisions or ledges increases. Instar I has 2 excretory ducts, older instars, including the prepupa, have 3 excretory ducts on each terminal process: apically, medially, and basally. As the larva matures (beginning with instar III), ledges or subdivisions begin to appear along the mesal (internal) margin of the terminal process and the excretory ducts become deeper and more defined (Figures 11e, 11h, 11i, 12d–12f). The medial and basal excretory ducts do not extend laterad or posterad, but are limited to the internal margin (Figure 11h). Other species may have either the medial excretory duct greatly extending laterad with the basal excretory duct confined to the internal margin, for example as in *A. subcinctus* Gory [41], or both excretory ducts greatly extending laterad as in *A. anxius*, *A. biguttatus* (Figure 5g), and *A. politus*. Whether the excretory ducts greatly extend laterally in *A. guerini* remains unclear as we only examined a slide mounted larval preparation of this species. However, superficially, the terminal processes of this species resemble the terminal processes present in instar III of *A. planipennis*. In *A. guerini*, the medial and basal excretory ducts are more pronounced and extend slightly laterad; furthermore, the entire process is not cylindrical but laterally compressed. All species examined, except *A. planipennis*, have shorter and stouter terminal processes with the apical excretory duct being moderately wide and lack the numerous subdivisions or ledges present in instars III, IV and prepupa of EAB.
6. Variations on the shape and pilosity of the labrum are highly informative also in delimiting assemblages above the species level (e.g., *A. viridis* and *A. ater* assemblages). In addition to the presence or absence of pilosity on the anterior margin of the labrum between the *A. viridis* and *A. ater* assemblages, the overall shape of the labrum also differs between these assemblages. Species in the *A. ater* assemblage have slight lateral expansions directly beyond the apex of the palatine sclerites, making the anterolateral margin of the labrum, for species in the *A. ater* assemblage, subapically produced (Figures 3b, 3d, 3e), while for species in the *A. viridis* assemblage it is uniformly rounded (Figures 3a, 3c). The shape of the anteclypeus differs slightly among species (Figures 3a–3g), however, no specific pattern was apparent for these assemblages.
7. The labium is very useful in distinguishing among *Agrilus* species and features of this structure were used extensively by Alexeev [30], [32] in his keys and descriptions of larvae of Palearctic *Agrilus* (Figures 1b, 4a–4g, 8b–8e). *Agrilus planipennis* has a sinuate, almost zigzag posterior contour of the microsetal area and the space between the anterior margin of the labrum and the posterior border of the microsetal area is equal to approximately 1/3 of the distance from the anterior margin to the bases of the apical setae (Figures 1b, 4a, 8b–8e). This “Alexeev ratio” [29] varies among species and can be defined as the distance between the anterior margin and posterior border of the microsetal area over (/) the distance between the anterior margin and the bases of the apical setae of the corner sclerites of the prementum (Figure 1b).

A species-assemblage-level character found on the labio-maxillary complex is either the presence of microspinulae concentrated subapically on the mala and internal surface of the stipes and cardo (i.e., *A. viridis* assemblage) (Figures 4c, 4g) or a dense covering of microspinulae on the internal surface of the maxillae (i.e., *A. ater* assemblage) (Figures 4d–4f).

8. Mandibles of *A. planipennis* have well-defined apical teeth, lacking in *A. politus* (*A. viridis* assemblage) and *A. australasiae* (*A. ater* assemblage), which have smaller, numerous blunt teeth (*A. politus*; Figure 7h) or a completely smooth margin. The shape of the mandibles of *A. planipennis* and *A. politus* is very similar, being deltoid, while for *A. australasiae* the mandibles are quadrate to subquadrate. The penicillum in *A. planipennis* and *A. politus* is large, a characteristic typical of borers feeding on hard wood. However, the structure of the apex, cutting edge, and the shape of mandibles appears related to the density of the larval food [43]; being adaptive characters and not necessarily indicative of phylogenetic relationship.
9. Spiracles of *A. planipennis* are more circular and complete (thoracic spiracles more “closed” than abdominal spiracles) than in *A. australasiae* [24].
10. The last segment of the antenna in *A. planipennis* is quadrate, while in *A. australasiae* it is deltoid. However, all sensory structures are present in both species with minor differences in position and size of microspinulae, located laterally and smaller in *A. planipennis* (Figure 7l) and apically and larger in *A. australasiae* [24]. Instars I and II of *A. planipennis* lack the fringe of microspinulae around the apex of antennal segment 1.

*Agrilus planipennis* larvae are recognized by the following combination of character states, including the first 6 states, which are unique among the species examined: 1, trapezoidal abdominal segments; 2, segment 10 setation sparse; 3, narrow, cylindrical terminal processes; 4, with numerous ledges appearing after instar II; 5, zigzag posterior contour of the microsetal area on prementum; 6, space between the anterior margin of prementum and posterior border of microsetal area is equal to approximately 1/3 of the distance from the anterior margin to the bases of the apical setae; 7, terminal processes with 2–3 excretory ducts; 8, smooth area between microdenticles and pronotal and prosternal grooves lacking; 9, pronotal groove posteriorly bifurcating; 10, prosternal groove entire; 11, labrum glabrous with margin not produced anterolaterally; 12, microspinulae concentrated subapically on the mala and internal surface of the stipes and cardo; 13, mandibles deltoid with well-defined apical teeth and large penicillum; 14, antennal segment 2 quadrate.

### Differences among instars

Minor differences exist between instars of *A. planipennis* [27], including the degree of pigmentation of sclerotized structures such as mandibles, as well as setation and relative size. The developmental stages can be distinguished by the number of excretory ducts making up the terminal processes (2 in instar I and 3 in instars II, III, IV+prepupa) and the presence (instars III, IV+prepupa) or absence (instars I+II) of ledges. Among instars I-IV, the ventral antero-lateral setae of the labrum do not increase in size, therefore the relative size of the setae decreases with each instar (Figures 2a–2h). Differences also exist in the thoracic and abdominal compression [compression of the prepupa, being much shorter than instar IV (Figures 14a, 14b)] and the subsequent curling of the prepupa, becoming J-shaped, which is a major behavioral difference. The shape of the microspinulae differs among instars I+II and III+IV+prepupa, having comb-like microspinulae in the former assemblage (Figures 12a–12c) and differentiated (single) microspinulae in the latter (Figure 9f).

Variation in the size and shape of the following structures has been used to determine the number of larval instars and duration of stadia for *A. planipennis* and other *Agrilus* larvae: terminal processes (frequently referred to as urogomphi), prothoracic plate, body width and length, and epistome width/length ratio (erroneously referred to as peristome [27]).

### Conclusion

This study upholds the segregation of *Agrilus* larvae into two assemblages based mainly on differences in the mouthparts, the *A. viridis* and the *A. ater* assemblages as proposed by Volkovitsh & Hawkeswood [24]. Based on features of the larvae, retention of *A. planipennis* in the subgenus *Uragrilus*, which includes also *A. ater* and *A. guerini* as suggested by Alexeev [22], [29], is dubious and substantiates recent studies [4] suggesting *A. planipennis* to be most closely related to species in the *A. cyaneoniger* group based on characters of the adult. However, that hypothesis could not be explicitly addressed in this study since immature stages of those species remain unknown.

While *A. planipennis* shares a similarly shaped posteriorly bifurcated pronotal groove with *A. biguttatus*, they differ in key characters, mainly the mouthparts and terminal processes. Even though larvae of *A. planipennis* are more similar to those in the *A. viridis* assemblage than to those in the *A. ater* assemblage (where species of *Uragrilus* cluster), adult characters do not support the placement of *A. planipennis* in the subgenus *Agrilus* where *A. viridis* and *A. politus* are currently classified based on adult characters. As such, given the limited knowledge of immatures in the genus (described for approximately 50 species) and pending a comprehensive phylogenetic analysis, this arrangement of classifying larvae into two major assemblages is for utilitarian purposes and not necessarily a reflection of evolutionary history.

Accurate identification of all life stages is essential to detect and successfully control and contain the spread of invasive forest pests like *A. planipennis*. Sets of characters herein described and illustrated will form the basis for future studies aimed at understanding the phylogeny of *Agrilus*. Understanding the evolutionary history of a group of organisms allows scientists not only to make predictions about potential invasive species with similar evolutionary histories and adaptations, but also helps scientists determine ways to manage invasive pests.

## Materials and Methods

Terminology follows Volkovitsh [43] and Volkovitsh & Hawkeswood [24], [44] with minor modifications (Figures 1a–1d). Explanation for some of the terms used for cuticular and sensory structures is provided:

Microspinulae (Figures 1d, 6d, bottom, 9b, 9e, 12a–12c): minute, cuticular outgrowths or spines with wide base, not or poorly sclerotized and not articulated to cuticle, without sensory function; sometimes reduced (Figure 9b, right); variable in size, length (tubercle-like–setiform; Figure 9e), and arrangement (singular–comb-like; Figures 6b, 12a). We regard microspinulae to be basic types of cuticular structures that can transform into microdenticles and asperities.

Microdenticles (microteeth) (Figures 5a–5d, 6d, top, 9c, 9f, 9h, 10e): short, broad, triangular, heavily sclerotized and frequently pigmented; usually located on sclerotized tubercles; sometimes denticles/sclerotized apex reduced or rubbed down and only sclerotized tubercles remain (Figures 6d, top, 9b–9d, 10e).

Microsetae (Figures 1b, 8c, 8e): articulated to cuticle, very short and thin setae (usually forming microsetal area on labrum and prementum), moderately sclerotized, presumably also without sensory function, situated usually on mouthparts.

Palmate sensillae [29] (Figures 1d, 7j–7l): pair of sensillae with digitiform apical outgrowths situated close to base of sensory appendage on top of the 2^nd^ segment of antennae.

Corner sclerites of prementum (Figures 1b, 8h): sclerites at latero-basal corners of prementum bearing apical setae and campaniform sensillae. Presumably, rudiments of labial palpi.

Homology of terminal processes with urogomphi is unwarranted. Urogomphi are derivates of the 9^th^ abdominal segment [45], [46] while terminal processes are located on the 10^th^ segment. Terminal structures present in *Agrilus* should be termed terminal processes and are continuous with the 10^th^ segment. In some Buprestidae species, these terminal processes are present only in neonate larvae and lost in the mature larvae (*Buprestis* Linnaeus) [47] or they are present in all the larval instars and lost only in the prepupa (*Anocisseis* Bellamy) [48]. In Aphanisticini and *Ethonion* Kubáň there is a pair of lightly sclerotized tubercles instead of processes on the 10^th^ segment [24]. We consider terminal processes to be secondary ectodermal structures of the 10^th^ segment. Functionally, terminal processes serve to aid in the compression of excrements and as a support during larval movement within the galleries, as such, forming a morpho-functional complex with shortened VIII–X abdominal segments [29].

Abbreviations (codens) for institutions and collections used in the text follow Evenhuis [49]:

NMNH–National Museum of Natural History, Washington, DC, USA.

NMPC–National Museum (Natural History), Prague, Czech Republic.

ZIN–Zoological Institute, Russian Academy of Sciences, St. Petersburg, Russia.

### Material examined

*Agrilus planipennis* Fairmaire: U.S.A. MI, Eaton Co., Potterville, Fox Memorial Park, October 12, 2010, T. M. Ciaramitaro [instars I–prepupa]; MI, Clinton Co., Bath, Private property on Ballentine Rd, April 1, 2010, T. M. Ciaramitaro [pupae and adults], 1–5 specimens of each stage. ex *Fraxinus pennsylvanica*. NMNH

*Agrilus* (*Uragrilus*) *anxius* Gory (bronze birch borer): U.S.A. #1664b, 2 specimens, instar III or IV. NMNH

*Agrilus* (*Uragrilus*) *ater* (Linnaeus): Russia, Belgorod region, Borisovka env., Forest on the Vorskla River Reserve, ex under the bark of dead trunk of *Populus* sp. (Salicaceae), August 1971, M. G. Volkovitsh. 2 specimens, instar III or IV. ZIN

*Agrilus australasiae* Laporte & Gory: Australia, New South Wales, Hastings point, ex dead, dry stems of *Acacia sophora* (Labill.) R. Br. (Fabaceae), October 1987, T. J. Hawkeswood, 2 specimens, instar IV. ZIN

*Agrilus* (*Anambus*) *biguttatus* (Fabricius): Russia, Belgorod region, Borisovka env., forest on the Vorskla River Reserve, ex dead trunk of *Quercus robur* (Fagaceae), August 1971, M. G. Volkovitsh, 2 specimens, instar IV. ZIN

*Agrilus* (*Uragrilus*) *guerini* Lacordaire: [Czech Republic] Mor. Paskov ex. *Salix viminalis* L. (Salicaceae) September 2 1990, J. Va’vra, 1 specimen, instar IV. NMPC

*Agrilus* (*Diplolophotus*) *nubeculosus* Fairmaire: Israel, Dead Sea Area, Nahal David, ‘En Gedi env., ‘En Gedi Reserve, ex dry branches of *Acacia* sp. (Fabaceae), 9 July, 1996, M. G. Volkovitsh and M. Yu. Dolgovskaya. 2 specimens, instar IV. ZIN

*Agrilus* (*Agrilus*) *politus* (Say): U.S.A. #13075a. 2 specimens, instar III or IV. NMNH

### Slide preparation

To study larval structures, microslides were prepared following the method used by Alexeev [29] using Fohr-Berlese media that acts also as a clearing agent to decompose soft tissue. Two slides were prepared per larval specimen: 1) mouthparts and 2) larval integument.

1. Mouthparts were separated from the head capsule along the posterior margin of the hypostome–pleurostome–epistome complex ( =  peristome; all apical sclerotized structures of the head) using dissecting microscissors (Fine Science Tools, Foster City, CA, USA). Cutting into sclerites was avoided during dissection. Once the mouthparts were separated from the head, the mandibles were “popped out” with a pin or sharp forceps by gently exerting lateral (external) pressure on the inner subapex of the mandibles. Once both mandibles were extracted, the peristome complex was separated by inserting a pin between the hypostome–epistome suture (pleurostome). Both antennae and labrum were retained with the epistome. Any remaining external tissue was then removed from all sclerotized parts. The Fohr-Berlese media was placed in the center of a clean slide in the shape of a cross. The dissected mouthpart sections were arranged with the external surface upwards and along the y-axis of the cross, starting with the pair of mandibles, then the epistome and last the hypostome+pleurostome. Four minute pieces of firm paper were placed at the corners of the cross to prevent damage of the mandibles by pressure. A glass cover slip, previously rinsed in alcohol and dried, was slowly lowered over the Fohr-Berlese preparation slide from the margin of the liquid to avoid creating bubbles in the medium. Additional Fohr-Berlese media was placed along the sides of the cover slip to fill any gaps. The medium was drawn under the cover slip.
2. After the mouthparts were separated, the larval body was cut along a pleural line from the thorax to approximately the 9^th^ abdominal segment. The head capsule remained intact. The body was placed into 10% KOH aqueous solution and boiled until soft tissues were dissolved and the integument became completely transparent (approximately 5–10 minutes). The transparent integument was rinsed three times in water. Fohr-Berlese media was placed on a cleaned slide and then the integument was positioned with the external surfaces of the dorsum and the venter facing upward. This step took several minutes because the integument often became twisted during the rinsing process. Working over a black background was found to be helpful. After the integument was completely extended, the cover slip was placed on the slide from the margin of the liquid and very gently and slowly lowered with forceps or a pin to avoid bubbles. The slides were continuously maintained in a horizontal position and then placed for a few hours in an oven at approximately 30°C.

### Imaging

The following equipment was used for observation and imaging: Larval body: a Leica (Wetzlar, Germany) MZ 9.5 dissecting microscope with a Leica DFC290 mounted camera. Instar IV slides: a Leica DME light microscope with a Panasonic (Secaucus, NJ, USA) Super Dynamic WV-GP460 analogous camera. Instar I: a Leica DM 5000 B polarizing microscope with a Leica DFC320 mounted camera. Instars II, III, prepupa, pupal body: a Zeiss (Oberkochen, Germany) Discovery.v20 stereomicroscope and an AxioCam HRc; mouthparts of instars II, III, prepupa: a compound microscope Leitz DIAPLAN with an AxioCam HRc. Scanning electron micrographs (SEM) were taken with a Philips XL-30 ESEM with LaB6 electron source.

Ideally, *A. planipennis* larvae would be compared to the larvae of species in the *A. cyaneoniger* species-group, which are hypothesized to be the closest relatives of *A. planipennis* based on adult features [1], [4]. However, immature stages of species in this group remain unknown (i.e., *A. agnatus* Kerremans, *A. auristernum* Obenberger, *A. bifoveolatus* Kerremans, *A. cyaneoniger* Saunders, *A. lafertei* Kerremans, *A. lubopetri* Jendek, *A. qinling* Jendek). For this reason, *A. australasiae*, described in detail by Volkovitsh & Hawkeswood [24] and 6 other distantly related species, were used for comparison.

### Animal ethics and research permit approval

All necessary permits were obtained for the collection of larval samples of *A. planipennis* in Fox Memorial Park in Potterville, MI, USA was approved by Mr Dan Patton, Director of Eaton County Parks and Ms Jackie Blanc Manager of Fox Memorial Park. Collection of samples from Bath, MI, USA was approved by the landowner, Mr John Valo. No specific permits were required for the collection of samples from the Forest on the Vorskla River field station in Russia in 1971, it was not privately owned or protected, and the field study did not involve endangered or protected species.
